# Influence of Polylactide (PLA) Stereocomplexation on the Microstructure of PLA/PBS Blends and the Cell Morphology of Their Microcellular Foams

**DOI:** 10.3390/polym12102362

**Published:** 2020-10-15

**Authors:** Zhiyuan Sun, Long Wang, Jinyang Zhou, Xun Fan, Hanghai Xie, Han Zhang, Guangcheng Zhang, Xuetao Shi

**Affiliations:** 1 Queen Mary University of London Engineering School, Northwestern Polytechnical University, Xi’an 710129, China; z.sun@se17.qmul.ac.uk (Z.S.); z.jinyang@mail.nwpu.edu.cn (J.Z.); 2 NPU-QMUL Joint Research Institute of Advanced Materials and Structures, Northwestern Polytechnical University, Xi’an 710072, China; W97L0115@163.com (L.W.); fanxun369199211@163.com (X.F.); xhh670995979@163.com (H.X.); han.zhang@qmul.ac.uk (H.Z.); 3 School of Chemistry and Chemical Engineering, Shaanxi Key Laboratory of Macromolecular Science and Technology, Northwestern Polytechnical University, Xi’an 710129, China; 4 School of Engineering and Materials Science, Queen Mary University of London, Mile End Road, London E1 4NS, UK

**Keywords:** PLA stereocomplexation, microcellular foaming, PBS, melt strength

## Abstract

Polylactide foaming materials with promising biocompatibility balance the lightweight and mechanical properties well, and thus they can be desirable candidates for biological scaffolds used in tissue engineering. However, the cells are likely to coalesce and collapse during the foaming process of polylactide (PLA) due to its intrinsic low melt strength. This work introduces a unique PLA stereocomplexation into the microcellular foaming of poly (l-lactide)/poly (butylene succinate) (PLLA/PBS) based on supercritical carbon dioxide. The rheological properties of PLA/PBS with 5 wt% or 10 wt% poly (d-lactide) (PDLA) present enhanced melt strength owing to the formation of PLA stereocomplex crystals (sc-PLA), which act as physical pseudo-cross-link points in the molten blends by virtue of the strong intermolecular interaction between PLLA and the added PDLA. Notably, the introduction of either PBS or PDLA into the PLLA matrix could enhance its crystallization, while introducing both in the blend triggers a decreasing trend in the PLA crystallinity, which it is believed occurs due to the constrained molecular chain mobility by formed sc-PLA. Nevertheless, the enhanced melt strength and decreased crystallinity of PLA/PBS/PDLA blends are favorable for the microcellular foaming behavior, which enhanced the cell stability and provided amorphous regions for gas adsorption and homogeneous nucleation of PLLA cells, respectively. Furthermore, although the microstructure of PLA/PBS presents immiscible sea-island morphology, the miscibility was improved while the PBS domains were also refined by the introduction of PDLA. Overall, with the addition of PDLA into PLA/10PBS blends, the microcellular average cell size decreased from 3.21 to 0.66 μm with highest cell density of 2.23 × 10^10^ cells cm^−3^ achieved, confirming a stable growth of cells was achieved and more cell nucleation sites were initiated on the heterogeneous interface.

## 1. Introduction

In recent years, polylactide (PLA), as an aliphatic biodegradable material derived from renewable resources such as corn and plant straw, has attracted exuberant research interests due to its good biocompatibility and degradation. It can be hydrolyzed primarily into oligomers with lower molecular weight under humid conditions then decomposed into H_2_O and CO_2_ by the digestion of micro-organisms presented in the environment. [1,2]. Related to its outstanding strength and high elastic modulus which are comparable to those of PP, PS, PE [3], PLA is considered as a potential candidate for replacing petrochemical polymers [4,5]. Many successes and products based on PLA can be found in the fields of biomedical pharmaceuticals and commodity packaging.

Nevertheless, the intrinsic brittleness and poor toughness are the prominent drawbacks for PLA [6]. Moreover, its low melt strength, low crystallinity and low thermal deformation temperature restrict its further application. In literature, the main strategies to improve the PLA’s properties are widely studied including copolymerization [7], blending [8], stereocomplexation [9], plasticization [10], chemical modification [11] composition with nanoparticles [12].

Recently, PLA stereocomplexation, which is based on the strong hydrogen bonding between poly (l-lactide) (PLLA) and poly (d-lactide) PDLA enantiomers and the consequential formation of complementary structures [13], is becoming one attractive method for improving the mechanical strength, thermal stability and gas barriers properties from neat PLA. The interaction between PLLA and PDLA polymers chains was assigned to be the hydrogen bond of CH_3_…O=C, by the investigation of the real time infrared spectroscopy during the isothermal melt crystallization process of the PLLA/PDLA in literature [14]. The melting point of PLA stereocomplexation crystals is about 230 °C, which is 50 °C higher than that of PLLA or PDLA homopolymer [15,16]. The applications of this unique PLA stereocomplexation are mainly in controlling stable micelles in drug delivery [17], tissue engineering scaffolds [18] and also acting as nucleating agents [16] in PLA modification.

Another strategy to overcome these limitations of PLA is by foaming process, to increase PLA′s crystallinity by the strong plasticization of blowing agent and the extensional flow during the foaming expansion process [19,20,21]. In fact, the microcellular polymers are foamed plastics characterized by a cell density greater than 10^9^ cm^−3^ and a fully grown cell size on the order of 10 μm. Compared with ordinary plastics, the quality of microporous foam material can decrease by 5–10% while maintaining high impact strength and low thermal conductivity [22].

However, one of the main challenges of PLA foaming is its low melt strength, resulting in the collapse or coalescence of the foams. Meanwhile, the sizes of cells and cell distribution are difficult to control during the foaming expansion process. Various research efforts have been made to overcome those drawbacks ranging from compounding with different types of additives such as cellulose fibers and chain extender [23,24,25,26], blending with other polymers having a higher crystallization rate [27], to controlling the gas content [28].

In this work, the unique PLA stereocomplexation mechanism was introduced in the PLA foaming processing with the aim of combining strengths from each strategy while overcoming their limitations by carefully designing the process. The crystals formed in the PLA matrix can promote cell nucleation through local stress variations and gas supersaturation [29], while the presence of considerable stereocomplex polylactide (sc-PLA) crystals with a high melting temperature can also increase the PLA’s melt strength through the enhanced molecular entanglement, which can suppress both cell coalescence and collapse [30].

The effect of foaming parameters on the crystal size was also considered and characterized in this work. Gas pressure is one of the key factors that affect the structure of crystals. A low CO_2_ pressure caused the defect of non-uniform foam and a large unfoamed area with high crystallinity and close-packed lamellas [31,32]. The CO_2_ can permeate into free volume aside polymer chains and improve the molecular chain mobility, which may induce the formation of a mesophase that promotes the crystallization of PLA [33,34]. Thus, the crystallinity can be adjusted by controlling the permeation of CO_2_ into the polymer matrix.

Bubbles growing around the crystal will cause deformation of the polymer chains and lamella because the rigid crystals can restrict polymer chain mobility. Xu et al. [35] reported that the high concentration of CO_2_ could induce the crystal form transition and increase the beta-phase crystal in the PVDF matrix. Li [36] firstly reported that CO_2_ could reduce the PLA interchain interaction while facilitating the polymer chains to migrate into crystal lattice during the spherulite growth process, which helped to form secondary lamella parallel to the original lamellas under high pressure instead of forming more branches. Therefore, to balance the mobility of the polymer chain and the crystallinity in matrix, high temperature and moderate pressure were used in this work.

Poly (butylene succinate) (PBS) was chosen as the second phase in the PLA matrix, which is a biodegradable material with promising toughness [37]. Furthermore, the microcellular foaming of the PLA/PBS was reported with a typical open-cell structure [38], which would be favorable for the application of PLA foams in the tissue engineering scaffolds with the requirement of high porosity for the cell migration and proliferation. Therefore, on the basis of introducing different proportions of PBS into PLA matrix, we also add PDLA to promote the formation of PLA stereocomplex crystals to improve the melt strength of PLA.

**Scheme 1:**
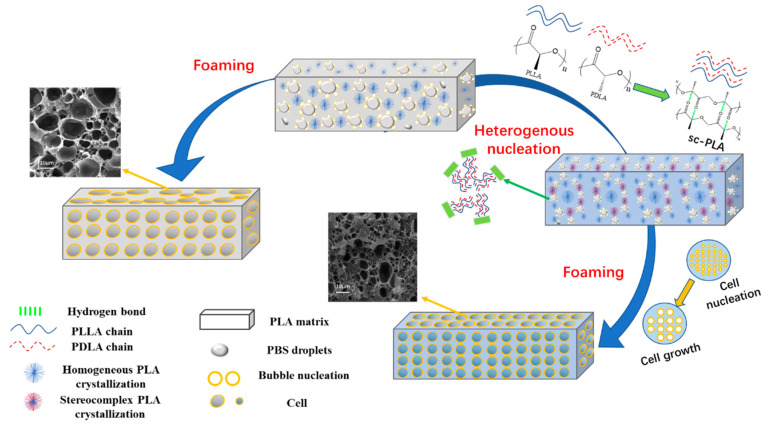
The formation process of polylactide (PLA) stereocomplexation and the improved cell growth in the foaming process.

In our former study [39], the formation of PLA stereocomplex crystal (sc-PLA) is strongly dependent on the ratio of PLLA to PDLA. The addition of PDLA with certain concentration (5–10 wt%) would also greatly improve the melt strength of PLA. This work investigated the influence of formed sc-PLA crystallites on dynamic mechanical properties and the rheological properties, the crystallization behavior of the ternary PLA/PBS/PDLA blends, then further regulate the foaming morphologies as shown in Scheme 1, which would widen the foaming processing window and applicable potential of PLA without compromising its biodegradability.

## 2. Materials and Methods

### 2.1. Materials and Samples Preparations

The PLLA (Ingeo 4032D) and PBS used in this work were pursued from Natureworks LLC (Minnetonka, MN, US) and Shandong Huiying New Materials Company (Zibo, China), respectively. The density of PLLA is 1.24 g/cm^3^, and the weight-average molecular weight (*M*_w_) is 194,000 g/mol. The melt flow index is around 3 g/10 min (190 °C/2.16 kg). Carbon dioxide with purity higher than 99.99% was pursued from Changte Air Product Co., Ltd. (Xi’an, China).

The PLA/PBS and PLA/PBS/PDLA blends were prepared by melting extrusion, after all the pellets were dried overnight. A twin-screw extruder (SHJ-95, Lantai Plastics Machinery Co, Ltd., Lanzhou, China) with a screw diameter of 21.6 mm and l/d ratio of 40 was used. The compounding temperatures of eleven zones of the barrel were set in the range of 150 and 220 °C with the screw speed set at 100 RPM. Table 1 records the sample list with different blending ratios of PDLA and PBS in the PLLA matrix used for foaming. The prepared PLA/PBS binary and PLA/PBS/PDLA ternary blends were compressive molded into sheets with a thickness of 0.5 mm at 190 °C for subsequent foaming processes. To investigate the influence of amount of PDLA on the viscosity, a mixer (HAAKE Rheomex 252 with PolyLab OS system, Thermo Fischer Scientific, Karlsruhe, Germany) was used to process the PLA/PBS/PDLA blends at 200 °C process temperature and 100 RPM rotating speed. The process mode was set as typical plastic melting and mix time was limited within 10 min to prevent the thermal degradation.

### 2.2. CO_2_ Saturation and Batch Foaming

The gas saturation process for PLLA sheets was conducted in a high-pressure vessel with the pressure and temperature kept at 15 MPa and at 120 °C. The high-purity CO_2_ was purged into the autoclave chamber by a supercritical fluid pump (SSI, S10SNXP1, LabAlliance, NY, USA) from the CO_2_ cylinder. The sample was kept in the vessel for 24 h to ensure the absorption equilibrium.

Then the saturated samples were immersed into silicon oil bath maintained at the foaming temperatures of 140 °C for 10 s to generate the microcellular foams. Then the foamed composites were immediately quenched into water, followed by drying process under a vacuum at 30 °C for 24 h.

### 2.3. Crystallization Behavior Analysis

The thermal behaviors of the PLLA and its blends were assessed with a differential scanning calorimeter (DSC1, Mettler Toledo, Switzerland). Hermetically sealed aluminum pans containing approximately 5 mg of the blend materials were used to place the samples in all the experiments. The samples were heated/cooled at a rate of 10 °C/min in the range from 25 to 250 °C. All samples were kept isothermal for 5 min to remove thermal history. The glass transition temperature (*T*_g_), cold crystallization temperature (*T*_c_), enthalpy of cold crystallization (Δ*H*_c_) and apparent melting enthalpy (Δ*H*_f_) were determined from the DSC curves.

The crystallinity of the PLLA phase was calculated by the following formula:

(1) $$X_{c}(\%crystallinity)=(\Delta H_{f}-\Delta H_{c})/(\Delta H^{0}(1-W_{f}))\times 100\%$$

where $\Delta H^{0}$ is the enthalpy of melting per gram of 100% crystallinity (perfect crystal) of PLA 93.7 J g^−1^) [40], and *W*_f_ is the weight fraction of PBS in the PLA/PBS binary blends.

The dynamic mechanical properties of PLA/PBS binary and PLA/PBS/PDLA ternary blends were studied by dynamic mechanical analysis (DMA) (TA Instruments, TA Q800, New Castle, DE, USA). The sheets used for DMA analysis were cut into a dimension of 6 × 40 × 0.5 mm^3^ and vibrated in tension mode at a frequency of 1 Hz. All the DMA tests were carried out from room temperature to 120 °C using a 2 °C/min temperature ramp.

### 2.4. Foam Morphology Characterization

PLA/PBS foams with or without PDLA were immersed in liquid nitrogen and then immediately fractured. The exposed surfaces were sputter coated with Au/Pd. Scanning electron microscopy (SEM, TESCAN (VEGA 3 LMH), ActipôleSt Charles, France) was used to investigate microcellular morphologies of the prepared foams. The SEM images were conducted using the software Image-Pro Plus to obtain the average cell size and cell density. The cell density *N*_f_, defined as the number of cells per cubic centimeter of the foam, was calculated using the following equation [41]:

(2) $$N_{f}={\left[\frac{nM^{2}}{A}\right]}^{\frac{3}{2}}$$

where *n* is the number of cells, *A* is the area of the micrograph in cm^2^, and *M* is the magnification factor.

Transmission electron microscopy (TEM, FEI-Talos F200X, Hillsboro, OR, USA) was used to investigate the distribution of PBS droplets in the PLLA matrix. The extruded sample was cut into 100 nm thick sample pieces then the thin pieces were placed on the copper network, finally the phase distribution was observed at 200 KV voltage.

## 3. Results and Discussion

### 3.1. Mixing Torque of the Blends

**Figure 1:**
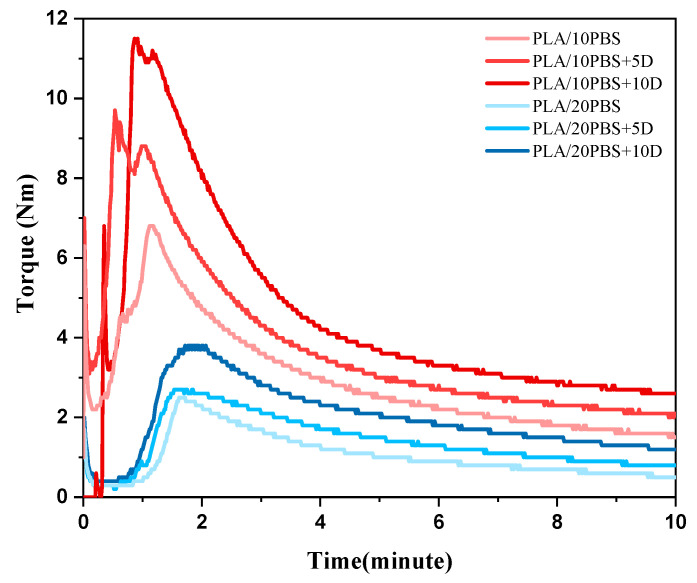
Mixing torque of the PLA/PBS blends with or without PDLA.

The influence of the introduced PDLA on the mixing torque of PLA/PBS was investigated during blending in a HAAKE internal melt mixer. As shown in Figure 1, the torque values of all the PLA blends increased remarkably during sample addition, and then they decreased and later gradually be equilibrium. The measured torque followed the typical melting and fusion process of polymer blends with a rapid increase followed by a decrease, with the final equilibrium state to indicate the formation of a stable melt. Notably, the torque dramatically reduced from 10 Nm to 2.5 Nm when the content of PBS increased from 10 wt% to 20 wt%, implying the flexible second phase impaired the melt strength of the PLLA matrix significantly. Conversely, the addition of PDLA led to an increase of torque, as PLA/10PBS+10D presented higher torque values than that of PLA/10PBS+5D and PLA/10PBS, where the same pattern was also found for PLA/20PBS+10D. These changes in the rheological properties could be evidence of the PLA stereocomplexation. More important, a higher PDLA amount would lead to higher amount of stereocomplex PLA crystals, resulting in a strong intermolecular interaction between PLLA and PDLA. The formation of strong physical pseudo-cross-link points between PLLA and PDLA would also affect the microstructure of PLA/PBS blends and the related crystallization.

### 3.2. Crystallization Behavior

**Figure 2:**
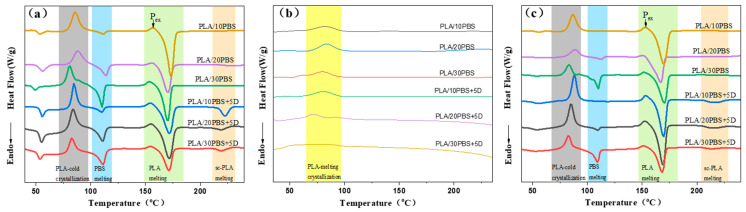
DSC curves of PLA/PBS blends with or without PDLA (**a**) the first heating curves (**b**) the first cooling curve (**c**) the second heating curves.

To improve PLA melt strength and hence its foamability, one strategy is to promote its slow crystallization behavior and crystallinity. However, an excessive level of high crystallinity with larger spherulite size and more chain-folded regions would lead to a dramatic decrease of gas absorption of supercritical blowing gases, limiting the further foaming capability. Therefore, the influence of PLA stereocomplexation on the crystallization of the PLA/PBS blend is crucial for the subsequent microcellular foaming. In this work, the influence of the second PBS phase on the PLA’s crystallization behavior is firstly investigated based on the contents of PBS. As shown in Figure 2a, when the concentration of PBS increases from 10 wt% to 30 wt%, the cold crystallization temperature slightly shifted to a lower temperature alongside with a minor reduction of cold crystallization enthalpy, implying the crystallization process had occurred earlier. In our previous study [38], the PBS droplets were uniformly distributed in the immiscible PLA/PBS blend and acted as “island-like” domains. Therefore, the introduced PBS phase initiates the heterogeneous nucleation and optimizes the crystallization behavior of PLA. The calculated PLLA crystallinity in Table 2 present slightly increase when more PBS was added into PLLA matrix. This phenomenon is consistent with the study of PLA/PBS blends in the literature [42]. Besides, the introduction of PBS has improved the chain mobility of PLA due to the high flexibility of the PBS phase, resulting in a slight decrease of *T*_g_ of the PLLA matrix. With the addition of PBS, the *T*_g_ of binary blends decrease from 51.1 °C (PLA/10PBS) to 47.1°C (PLA/30PBS). After the addition of PDLA, the glass transition of ternary blends slightly increases compared to that of their related binary blends, which could be related to the limited chain mobility due to the formation of sc-PLA. Another phenomenon that should be noted is the multiple melting behavior of PLA/PBS blends [43]. The transition of α′-form crystals to their α-counterparts occurred before the PLA/PBS melted, which is characteristic by a small exothermal peak prior to the dominated melting peak, marking as P_ex_. This α′-α transition could be caused by the quenching process from the compounding temperature of 200 °C since the α′-form crystals are produced in the crystallization at low crystallization temperature as explained in the literature [44,45].

Furthermore, the effect of 5% PDLA on the crystallization behavior of PLLA was also investigated. As shown in Figure 2a, after the addition of PDLA, a stereocomplex peak appears in a range from 220–230 °C, which is about 50 °C higher than the *T*_m_ of PLLA homopolymers. The presence of *T*_sc-m_ proves that the PLA/PBS/PDLA ternary blends contains stereocomplex crystallization. This phenomenon is also consistent with the torque curve of the HAAKE internal melt mixer. In the literature [46] and our form work [47], certain amounts of added PDLA could significantly improve the crystallization rate and crystallinity of PLLA homopolymer by virtue of the heterogenous nucleating effect of stereocomplex PLA. However, these works were normally investigated on the PLLA/PDLA systems without other compositions.

With the addition of PDLA into the PLA/PBS, the cold crystallization peak (grey part in Figure 2a) appears to be remarkable at a relative higher temperature region. Moreover, the calculated crystallinities of PLLA in the ternary blend (Table 2) present an obvious decrease with the addition of 5% PDLA. This could be explained by the possibility that the PLLA is more inclined to form sc-PLA network with 5 wt% PDLA, and the formed networks act as physical crosslinking points, which constrains the mobility of PLLA chains. In other words, only suitable PDLA concentration would refine PLLA crystallization behavior, while too much PDLA weakens the PBS effect and the PLLA chain mobility. From another perspective, the amount of PBS also influences the crystallinity of sc-PLA as shown in Table 2, which is related to the hydrogen bond formed between PLLA and PDLA. More PBS domains in the blends, less intermolecular action between PLLA and PDLA, leading to slightly decreasing sc-PLA crystallinity.

The DSC cooling curves and the related explanation are shown as Figure 2b. In a binary PLA/PBS blends, when the PBS concentration increases, the crystallization peak in the cooling process tends to be obvious. However, with the further addition of PDLA, the crystallization peak in the ternary blend presents much smaller area, which again confirms the decreasing crystallization due to the limitation effect on chain recruitment caused by the formed sc-PLA crystals.

In the DSC curves of the second heating in Figure 2c, similar trend of the cold crystallization peaks with the addition of 5% PDLA reflects the inhibited effect of PDLA (the consequence formed sc-PLA crystals) and PBS on the PLLA crystallization behavior. The influence of PDLA on the PLLA spherulites was also discussed in supporting information as Figures S1 and S2. Because of the existing sc-PLA with high melting temperature, both the PLLA and PBS crystals formed during the cooling process were shown in Figure S2a,b. The existed sc-PLA crystals also result in smaller PLLA spherulites.

According to the above results, the addition of both PBS and PDLA would constrain the chain mobility of PLLA, leading to a decrease in PLLA crystallinity. This would be favorable for the gas absorption of supercritical gases in the PLLA amorphous region, because it is a consensus that the well-organized chain-packing crystallites with strong molecular forces resist the supercritical gas to permeate [48]. In other words, the blowing agents prefer to solve in the free volume in the amorphous region even between the lamellar chain-folded chains in a spherulite. Another question for this PLA/PBS blends with PDLA is about the number of interfaces formed around the PBS domains or sc-PLA crystallites. In literature, the reserved sc-PLA crystals in the PLLA matrix due to the about 50 °C difference of their related melting temperature were reported to be from about 20 nm to 1μm mainly depending on their amount [39]. The existing interfaces were reported to have acted as gas reservoir sites. More interfaces represent more absorption gases in the system, and more cell nucleating sites for the foaming process. The influence of the PDLA or in other words the reserved sc-PLA crystallites on the microstructure of PLA/PBS is of importance for future foaming behavior.

### 3.3. Effect of Poly (d-Lactide) (PDLA) on the Microstructure of Polylactide (PLA)/Poly (Butylene Succinate) (PBS) Blends

**Figure 3:**
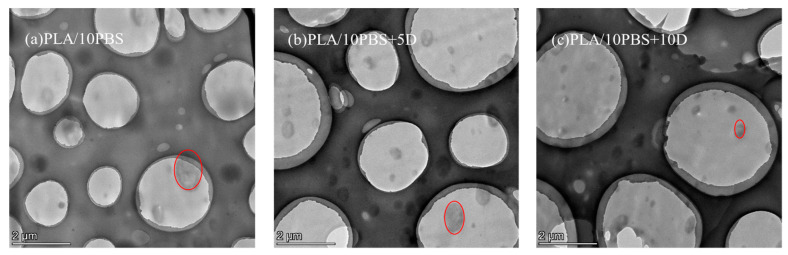

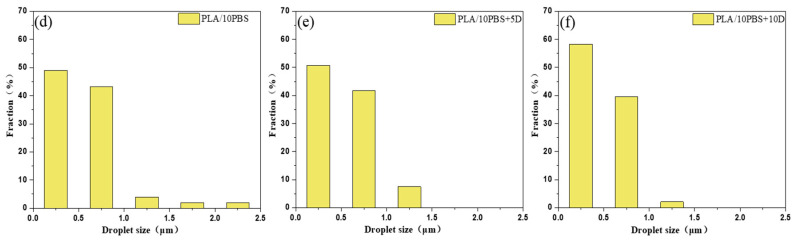
Transmission electron microscope (TEM) images for PLA/PBS blending with and without PDLA with scale bar in 2 μm (**a**) PLA/10PBS; (**b**)PLA/10PBS+5D; (**c**)PLA/10PBS+10D; (**d**) droplet size distribution of PLA/10PBS; (**e**) droplet size distribution of PLA/10PBS+5D; (**f**) droplet size distribution of PLA/10PBS+10D. (The red circles represent PBS domains.)

Figure 3 presents TEM photographs and droplets size distribution of PLA/PBS blends with different PDLA contents. In the PLA/10PBS blends, the dispersed PBS phase (shown with red marks) presented a granular distribution and when the content of PDLA increased from 0% to 5% to 10%, the average size of PBS droplets tended to be reduced from 0.603 μm to 0.527 μm to 0.459 μm. Moreover, the bar charts show that the droplet size was more uniform after PDLA was added, indicating that the addition of PDLA is beneficial for the distribution of PBS in the PLLA matrix. The similar results could be observed by the POM images in Figure S1.

The reason is mainly related to the formation of sc-PLA crystal after PDLA added into the incompatible PLA/PBS system. In literature, the sc-PLA crystal acted as a physical crosslink point that exists in a solid form [49], playing a similar role of particles inducing continuous phase transformation [50]. Due to the large incompatibility between the PLA and PBS, the PLLA/10PBS in this work showed no transition from the island-like phase to continuous phase, but exhibited a trend of refinement of second phase particles. This will also affect the rheological properties, thermal properties and foaming performance.

### 3.4. Effect of PBS on the Dynamic Mechanical Properties of PLA/PBS Blends

**Figure 4:**
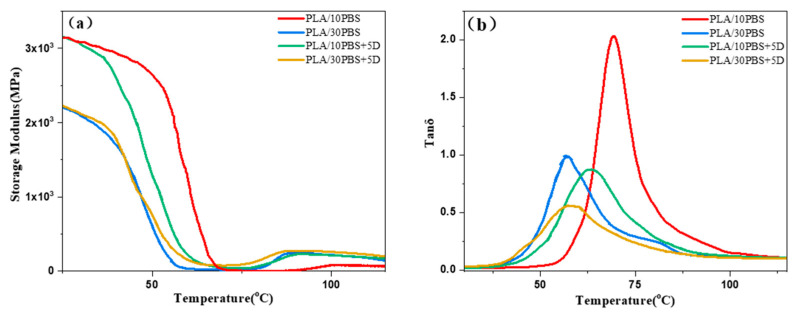
Temperature dependence of (**a**) the storage modulus and (**b**) tanδ for PLA/PBS blends with or without PDLA.

This work used dynamic mechanical analysis to study the viscoelastic properties of PLA/PBS blends with and without PDLA under the glass-transition temperature. Figure 4a illustrates the storage moduli change of PLA/10 PBS and PLA/30 PBS blends and ternary blends with 5 wt% content of PDLA. The storage moduli curves of PBS/sc-PLA blends exhibit similar variation trend via temperature, all curves dwindle at the beginning while descending significantly around 60–70 °C. This plunge is elicited by strain softening when the temperature surpassed the glass transition temperature. The polymer chain movement lags behind the change of external force during the heating process, and this hysteresis generates a large energy loss. When the temperature is over 85 °C, a slight increment appears on the storage moduli curves except the PLA/10PBS blends due to the enhanced mobility of PLA chains and cold crystallization process. The increasing storage moduli at higher temperatures are consistent with the above DSC results. Also, it can be found that with the increase of PBS content, the storage moduli of the PLA blends in glass state tend to decrease. This may be because the elastic deformation is more prevalent in the blend with the high content of PLLA, so the storage modulus is closer to the elastic modulus. With the further increase of the PDLA into the PLA/PBS blends, the storage modulus is slightly decreased. This could be explained by the former DSC results that indicated the decreasing of crystallinity.

Figure 4b exhibits the temperature dependence of the tanδ curves of PLA/PBS and PLA/PBS/PDLA specimens. The temperature at the peak corresponds to the glass transition temperature while it is slightly higher than the result of DSC. It can be seen that the area of loss peak decline with the increase of PBS content. Because when the temperature rises to 71 °C, the dynamic glass transition temperature of pure PLLA [51], the PLA chains just get out of the frozen state and obtain mobility while PBS chains can freely move at this temperature. Therefore, more content of PBS will lead to a smaller loss peak. Another phenomenon observed is that after the addition of PDLA, the dynamic glass transition temperature moves to the lower temperature, which may account for the plasticization effect of the second phase of PBS in the PLLA matrix. Also, a conclusion obtained from the *T*_g_ reduction tend is that the enhanced miscibility of PLLA and PBS when the PBS concentration reaches 30% or 5% PDLA is introduced to the PLA/PBS blends.

### 3.5. Foaming Morphology of PLA/PBS and PLA/PBS/PDLA Blends

**Figure 5:**
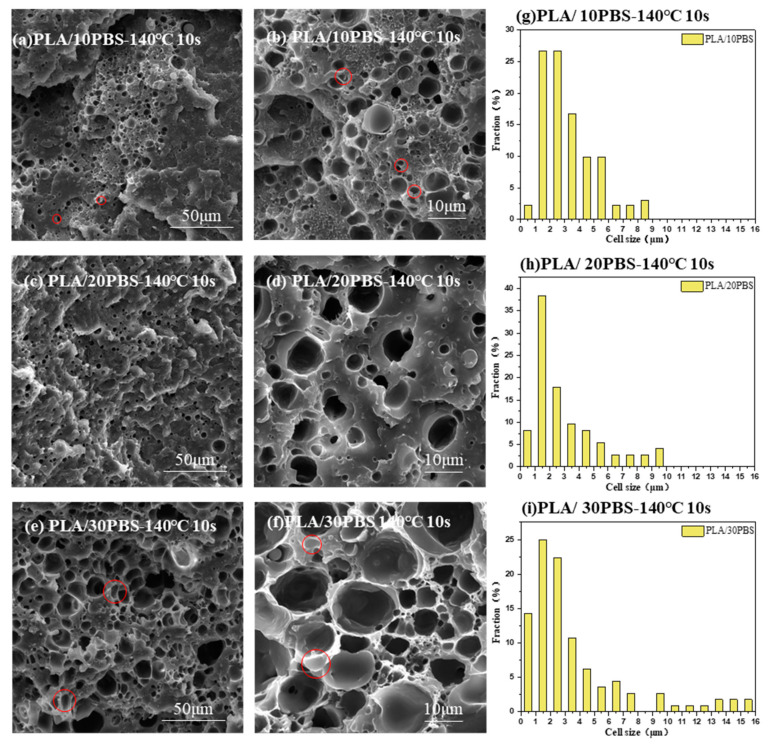
Scanning electron microscopy (SEM) images for PLA/PBS after foaming at 140 °C for 10 s. (**a**) PLA/10PBS 1000×; (**b**) PLA/10PBS 3000×; (**c**) PLA/20PBS 1000×; (**d**) PLA/20PBS 5000×; (**e**) PLA/30PBS 1000×; (**f**) PLA/30PBS 3000×; (**g**) cell size distribution of PLA/10PBS; (**h**) cell size distribution of PLA/20PBS; (**i**) cell size distribution of PLA/30PBS. (The red marks represent shaped PBS phase during foaming.)

Figure 5 shows the morphology of samples with different proportions of PLA/PBS binary blends after foaming at 140 °C. From Figure 5a,b, the SEM images with 1 k and 3 k magnification show the morphology of PLA/10PBS foams. It can be seen that a small content of PBS phase was distributed in the PLLA matrix in droplets manner while it did not exhibit an evident trend to drop from the matrix, which proved that PBS and PLLA were partially miscible. In addition, with the increase of PBS proportion, the white area tended to be more visible, even when the PBS accounts for 30%, the PBS phase still separated as domains without the presence of continuous phase. As PBS is more flexible than PLLA, blending PBS not only contributes to heterogeneous nucleation during the crystallization process, but also to the growth of foam cells.

In the condition of foaming at 140 °C for 10 s, the PLA/10PBS and PLA/30PBS both exhibited good foaming performance and some PBS droplets had a crescent shape with some cavities surrounded, as shown in Figure 5 with red circular marks. Since the initial structure of pure PLLA granules is closed, it is very challenging to prepare PLA foams with open cell structures without collapse and coalescence. The melting point of PBS granules used in this experiment is about 110 °C, thus PBS phase had already been flexible during the partially foaming temperature. Moreover, due to poor miscibility of PBS and PLLA, the CO_2_ adsorption and cell growth mostly occurred in the interface between PBS and PLLA phases. Consequently, the gas tended to diffuse to the softer area and applied stress to PBS domains during the growth of cells until the cell expands to a stable size, leaving crescent-shaped PBS granules and tensile fibers. That is the basic foaming mechanism of the PLA/PBS binary blends.

Figure 5c–f show the PLLA cell morphology with 20 wt% and 30 wt% PBS, the average cell size increased and the cell wall became thinner with the incremental area of PBS domains. This phenomenon is mainly on account of the formation of abundant bubble nuclei under heterogeneous nucleation during bubble nucleation. Since the PBS phase disperses in the PLA matrix in the micrometer scale, and the interface between PBS and PLA possesses lower activation energy for bubble nucleation and provides favorable heterogeneous nucleating sites for cell formation. Besides, with the help of PBS’s intrinsic flexibility, the toughness of the PLLA matrix has been well improved, which also contributes to the supersaturated gas diffusing to bubble nuclei and the growth of cells, resulting in larger cell size.

**Figure 6:**
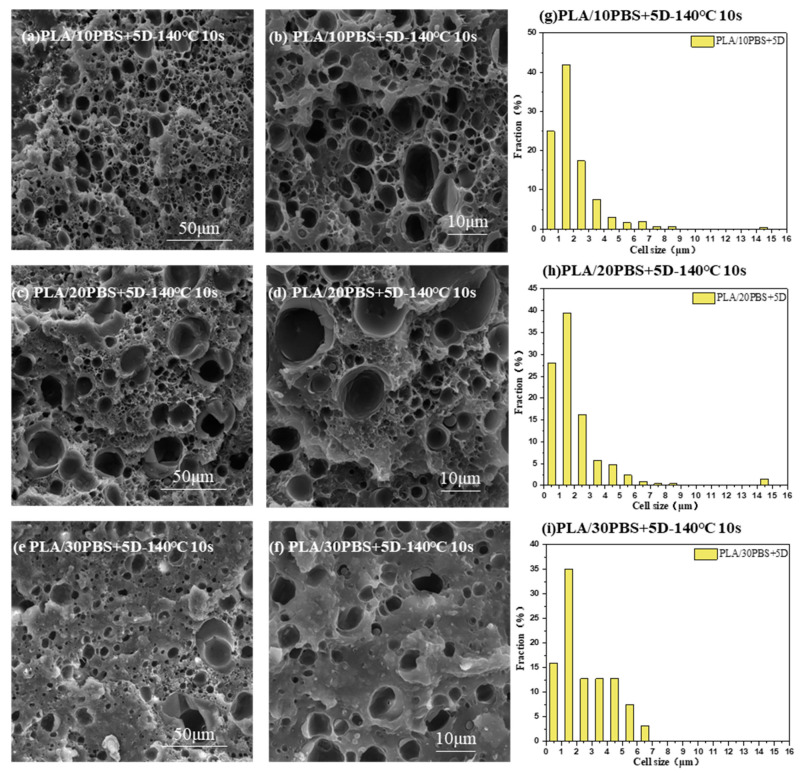
SEM images for PLA/PBS/PDLA after foaming at 140 °C for 10 s. (**a**) PLA/10PBS+5D 1000×; (**b**) PLA/10PBS+5D 3000×; (**c**) PLA/20PBS+5D 1000×; (**d**) PLA/20PBS+5D 3000×; (**e**) PLA/30PBS+5D 1000×; (**f**) PLA/30PBS+5D 3000×; (**g**) cell size distribution of PLA/10PBS+5D; (**h**) cell size distribution of PLA/20PBS+5D; (**i**) cell size distribution of PLA/30PBS+5D.

However, the second phase PBS improved cell sizes but also caused the worse cell homogeneity. Figure 5g–i present the cell size distribution of PLA/PBS binary blends, it can be found from the bar charts that the cell sizes fluctuate more apparently with the increase of PBS content. To better balance the relationship between cell morphology and cell size, this work also investigated the effect on the morphology of introducing 5 wt% PDLA to PLA/PBS blends. Figure 6a–f is the SEM pictures of PLA/PBS/PDLA ternary blends. Compared with binary blends, PLLA matrix with 5 wt% PDLA exhibited more distinct open-cell structures and the surface remained some ductile fracture characteristics. Moreover, the average cell size of PLA/10PBS with 5 wt% PDLA and PLA/30PBS with 5 wt% PDLA tended to reduce while the cell wall of PLA/30PBS with 5% PDLA became thicker. This change was beneficial for the control of foam homogeneity and in favor of uniform cell size distribution of PLA/PBS samples with PDLA.

Moreover, Figure 6 indicates that the formed sc-PLA had an insignificant effect on the microstructure of PBS. As for PLA/10PBS blends, the cell density increases manifestly after the addition of PDLA. The average cell sizes corresponding to PLA/10PBS and PLA/10PBS with 5 wt% PDLA are 3.21 μm and 0.66 μm respectively, while the related cell density increased from 6.36 × 10^9^ cm^−3^ to 2.23 × 10^10^ cm^−3^ (shown in the Table 3). The reason for this phenomenon may be due to the stereocomplexation existed in the PLLA matrix. The PDLA introduced into PLA/PBS blends could connect with PLLA through hydrogen bonds and formed sc-PLA network, which can act as a nucleating agent increases the number of crystallized domains. Theses domains not only facilitate the adsorption of gas but also increase the interface area, and thus the edges of the crystals can provide more bubble nucleation sites and increase the cell density.

Furthermore, this phenomenon suggested that the introduction of PDLA and the formation of sc-PLA triggered a strain-hardening effect and improved the melt strength of the PLLA matrix, resulting in a reduction of the average cell size. Notably, the crystallinity slightly decreased in ternary blends when compared with the crystalline degree of PLLA in PLA/PBS blends, which is related to the formed sc-PLA extended randomly in all directions and constructed a physical crosslinked network.

Table 3 presents the cell density and average cell size of foaming samples at the same temperature after the same gas saturation process. Different from the tendency of PLA/10PBS blends, the cell density and cell size of 7/3 blends both decreased after the addition of PDLA. This may account for excessive PBS impairing the effect of PDLA. The hydrogen bond endows PLLA and PDLA to form sc-PLA crystals, which inhibited heterogeneous nucleation and undermined PLLA chain recruitment. Besides, lower gas adsorption and fewer nucleation sites in ternary system are another possible reason for the reduction in cell size and density. Overall, the addition amount of PDLA and PSB needed to be matched properly to well balance the crystallinity and melt strength. When the PBS content is constant, the addition of PDLA is conducive to the improvement of melt strength, while the higher PDLA and more sc-PLA crystallization will adverse to the foaming process of PLA.

## 4. Conclusions

In this work, the influence of second-phase PBS and sc-PLA on the crystallization, dynamical mechanical properties and foaming behavior of PLLA matrix was investigated systemically. When 5 wt% PDLA was introduced to PLA/PBS blending system, the crystallinity of PLLA were undermined. This impediment on nucleus formation and crystal growth may account for a physical pseudo-cross-link points formed inside the PLLA matrix which restrained the molecular chain mobility of ternary blends and impaired the nucleating effect of PBS.

Although 5 wt% PDLA hindered the crystallization behavior, it balanced the crystallinity and melt strength of the PLLA matrix well, which created a positive effect on the supercritical gas adsorption process and foaming process, respectively. The cell morphology of PLA/PBS and PLA/PBS/PDLA foams are found in the open-cell structure and the cells showed an evident growth trend to soft PBS droplets that were distributed as an immiscible phase. Compared to PLA/PBS binary foams, the average cell size of PLA/PBS/PDLA ternary blends decreased while the cell density increased with better distribution uniformity. The remaining sc-PLA crystallites performed as the cell nucleating agents and, therefore, the decreasing cell size enhanced the PLLA foaming behavior without cell coalescence. This work utilizes the PLA stereocomplexation mechanism into the microcellular foaming to enhance the related melt strength of the PLA matrix. Meanwhile, this work provides a basic understanding of the sc-PLA crystallites on the microstructure of PLLA blends with other polymers and then favors the foaming behavior.

## Figures and Tables

**Table 1:** Composition of PLA/poly (butylene succinate) (PBS)-based blends with or without poly (d-lactide) (PDLA).

Sample Name	PLLA/wt%		PBS/wt%		PDLA/wt%	
PLA/10PBS	90	90%	10	10%	0	
PLA/20PBS	80	80%	20	20%	0	
PLA/30PBS	70	70%	30	30%	0	
PLA/10PBS+5D	90	85.7%	10	9.5%	5	4.8%
PLA/20PBS+5D	80	76.2%	20	19.0%	5	4.8%
PLA/30PBS+5D	70	66.7%	30	28.5%	5	4.5%
PLA/10PBS+10D	90	81.8%	10	9.1%	10	9.1%

**Table 2:** Differential scanning calorimetry (DSC) parameters for crystallization of poly (l-lactide) (PLLA)/PBS and PLLA/PBS/PDLA in the first heating process.

PLA/PBS +PDLA	T_g_/°C	T_cc_/°C	△H_cc_ (J/g)	T_m-PBS_/°C	T_m-hc_/°C	△H_m-hc_ (J/g)	X_m-hc_ (%)	T_m-sc_/°C	△H_m-sc_ (J/g)	X_m-sc_ (%)
90/10	51.1	85.5	17.9	111.2	172.3	43.2	30.0	-	-	-
80/20	50.8	86.7	14.5	113.6	170.1	37.6	30.9	-	-	-
70/30	47.1	80.6	12.2	110.0	169.9	33.3	32.1	-	-	-
90/10+5	53.8	84.5	19.8	110.1	171.6	36.0	19.2	219.6	9.7	7.5
80/20+5	53.2	83.6	18.7	110.7	171.3	34.5	21.0	213.5	6.6	5.8
70/30+5	51.3	82.7	14.6	111.1	171.0	27.2	19.2	213.0	4.9	4.9

**Table 3:** Cell characteristic statistics at 140 °C for 10 s.

PLA/PBS	9/1	8/2	7/3	9/1+5D	8/2+5D	7/3+5D
Cell Density/cm^−3^	6.36 × 10^9^	2.51 × 10^9^	5.04 × 10^9^	2.23 × 10^10^	1.30 × 10^10^	3.82 × 10^9^
Cell Size/μm	3.21	4.55	6.74	0.66	4.83	3.43

## Supplementary Materials

The following are available online at https://www.mdpi.com/2073-4360/12/10/2362/s1: Figure S1: Morphology of (a) PLA/30PBS blends. (b) PLA/30PBS+5D at 200 °C, Figure S2: Crystallization process of PLA binary and ternary blends.
