# Supplementary material for: Influence of Polylactide (PLA) Stereocomplexation on the Microstructure of PLA/PBS Blends and the Cell Morphology of Their Microcellular Foams
Source: Polymers (Basel). 2020 Oct 15;12(10):2362. doi: 10.3390/polym12102362 (PMC7602427; doi:10.3390/polym12102362)
Supplement: Supplementary file 1 [file polymers-12-02362-s001.pdf]

Supporting information to

Influence of PLA stereocomplexation on the microstructure of PLA/PBS blends and the cell morphology of their microcellular foams

Zhiyuan Sun[1,4], Long Wang[2,4], Jinyang Zhou[1,4], Xun Fan[2,4], Hanghai Xie [2,4], Han Zhang [3,4], Guangcheng Zhang[2]*, Xuetao Shi[2,4]*

To further investigate the influence of PDLA on the crystallization of PBS and PLLA, polarizing optical microscope (POM) was carried out used to record the crystallization process. To investigate the crystallization behaviour of PBS, the PLA/30PBS and PLA/30PBS+5D samples were chosen.

The specimens were heated to 230℃ and kept the temperature for 3 minutes to remove the thermal history, then quickly cooled to 40℃ to allow the crystal growth. Figure S1 exhibits the polymers blends morphology at 200℃. The white droplets in Figure S1 should be distributed PBS phase (circled in yellow). Comparing the Figure S1(a) and Figure S1(b), the droplet size of PBS in Figure S1 (b) was reduced, implying that the PDLA facilitated the uniform distribution of the second phase. This result was consistent with the TEM images in Figure 3 in the manuscript.

Figure S2 illustrates the crystal growth during a cooling process from 120℃ to 40℃ of PLLA/30PBS and PLLA/30PBS+5D samples. At 120℃, more PLA homogenous crystals (marked in yellow circle) appeared in the PLA/30PBS blends than that in the PLA/30PBS+5D, when temperature descended to 60℃, the size of spherulites in the former are much larger than the figure for crystals in PLA/30PBS+5D. Notably, three prominent spherulites(marked in red circles) appeared when the temperature decrease to 50℃. Those spherulites kept growing until those size reached a relatively stable value around 40℃. The appeared spherulites cycled in red were assigned to the PBS crystallization.

For the homogenous PLLA crystals in the ternary blends in Figure S2 (b) in the cooling process, the number of crystals in ternary blends were more than that in the binary blends. Besides, the dispersion of PLLA crystals in ternary blends was more uniform, implying that the PDLA and the

formed PLA stereocomplex crystals was beneficial for the crystallization as nucleating agents. Meanwhile, it is difficult to observe the related PBS spherulites at the temperature range from 50 to 40 ℃ because of the fully crystallized PLLA and limitation of observation. Combined with the results in Figure S1 and the DSC melting peak of PBS, the formation of PLA stereocomplex crystals would lead to the decrease of PBS spherulites size.

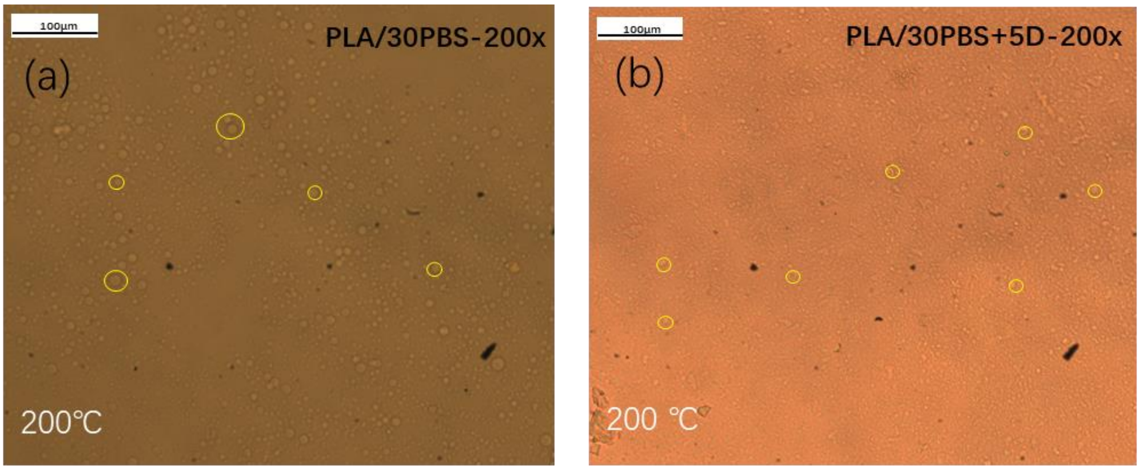

Figure S1.Morphology of (a)PLA/30PBS blends (B)PLA/30PBS+5D at 200℃

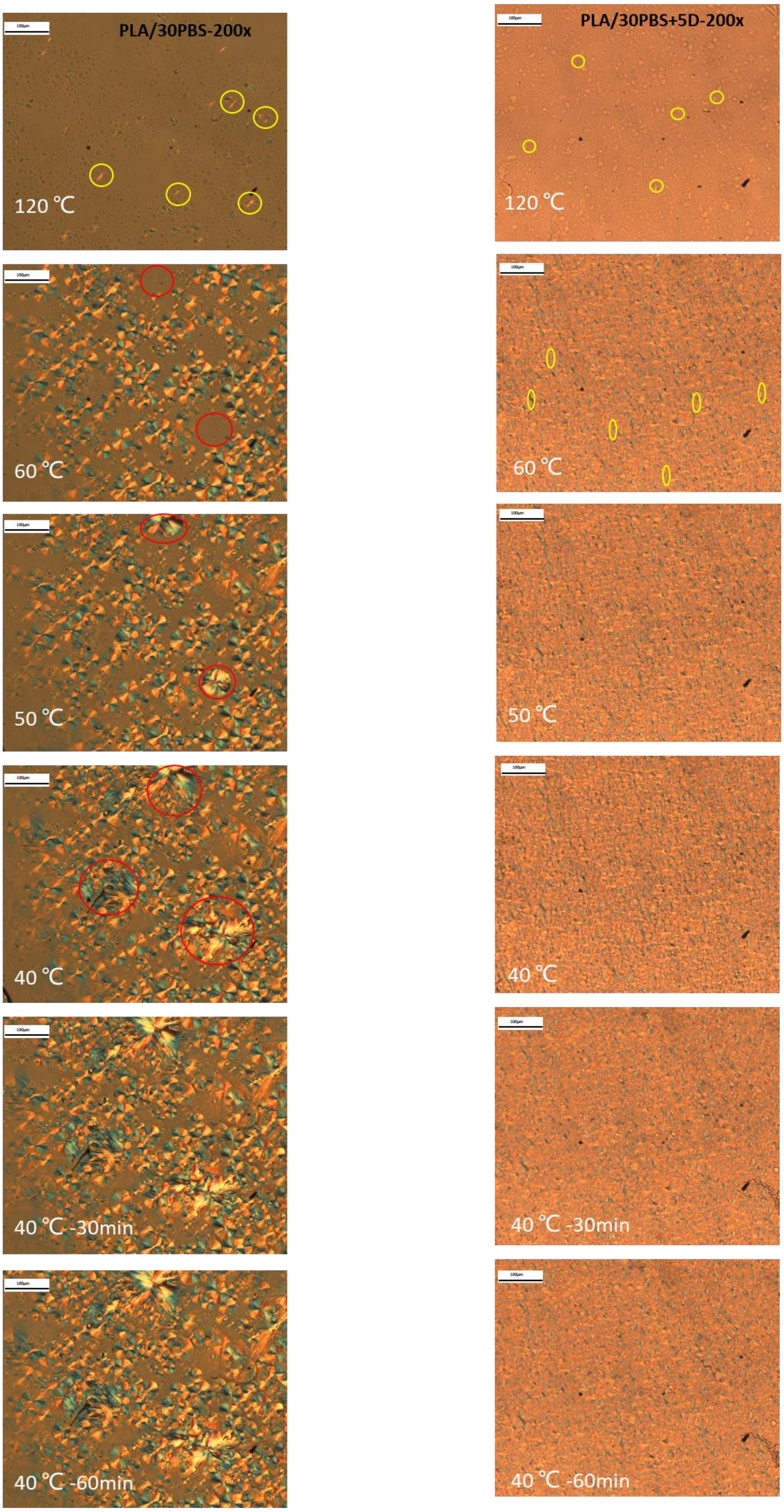

Figure S2. Crystallization process of PLA binary and ternary blends
